# Laboratory system strengthening and quality improvement in Ethiopia

**DOI:** 10.4102/ajlm.v3i2.228

**Published:** 2014-11-03

**Authors:** Tilahun M. Hiwotu, Gonfa Ayana, Achamyeleh Mulugeta, Getachew B. Kassa, Yenew Kebede, Peter F. Fonjungo, Gudeta Tibesso, Adino Desale, Adisu Kebede, Wondwossen Kassa, Tesfaye Mekonnen, Katy Yao, Elizabeth T. Luman, Amha Kebede, Mary K. Linde

**Affiliations:** 1 Ethiopian Public Health Institute (EPHI), Addis Ababa, Ethiopia; 2 US Centers for Disease Control and Prevention (CDC), Addis Ababa, Ethiopia; 3 International Laboratory Branch, Division of Global HIV/AIDS, US Centers for Disease Control and Prevention (CDC), Atlanta, United States; 4 The American Society for Clinical Pathology (ASCP), Chicago, United States

## Abstract

**Background:**

In 2010, a National Laboratory Strategic Plan was set forth in Ethiopia to strengthen laboratory quality systems and set the stage for laboratory accreditation. As a result, the Strengthening Laboratory Management Toward Accreditation (SLMTA) programme was initiated in 45 Ethiopian laboratories.

**Objectives:**

This article discusses the implementation of the programme, the findings from the evaluation process and key challenges.

**Methods:**

The 45 laboratories were divided into two consecutive cohorts and staff from each laboratory participated in SLMTA training and improvement projects. The average amount of supportive supervision conducted in the laboratories was 68 hours for cohort I and two hours for cohort II. Baseline and exit audits were conducted in 44 of the laboratories and percent compliance was determined using a checklist with scores divided into zero- to five-star rating levels.

**Results:**

Improvements, ranging from < 1 to 51 percentage points, were noted in 42 laboratories, whilst decreases were recorded in two. The average scores at the baseline and exit audits were 40% and 58% for cohort I (*p* < 0.01); and 42% and 53% for cohort II (*p* < 0.01), respectively. The *p*-value for difference between cohorts was 0.07. At the exit audit, 61% of the first and 48% of the second cohort laboratories achieved an increase in star rating. Poor awareness, lack of harmonisation with other facility activities and the absence of a quality manual were challenges identified.

**Conclusion:**

Improvements resulting from SLMTA implementation are encouraging. Continuous advocacy at all levels of the health system is needed to ensure involvement of stakeholders and integration with other improvement initiatives and routine activities.

## Introduction

Medical laboratories form the backbone of health systems, as test results are critical for diagnosing diseases, guiding treatment, determining drug resistance and identifying diseases of public health significance through surveillance.^1,2,3^ An integrated, tiered, functional and sustainable laboratory system is necessary in order to address these health system needs.^4^

Despite their importance, laboratories are often under-resourced, resulting in inadequate infrastructure, poorly-trained personnel and lack of standardisation.^1,2,5,6^ Likewise, laboratory services in Ethiopia received little attention until recent years. Funds and improved testing technologies were made available to laboratories after the HIV pandemic burdened medical facilities; however, several challenges to implementing laboratory improvement remain, such as the lack of adequately-trained personnel, clearly-defined responsibilities and well-established organisation.^7^

In response to these challenges, laboratory assessments were conducted on a national scale within Ethiopia so as to elucidate specific deficiencies; as a result, corrective measures were proposed. Amongst these were milestones of developing the first laboratory strategic plan in 2005 and mandating the Ethiopian Public Health Institute (EPHI) to lead laboratory programmes nationwide.^7^ The first laboratory strategic plan (2005 to 2008) was focused primarily on HIV, which accounted for the bulk of both testing and funding. This plan was used as a roadmap for implementing laboratory improvement programmes in Ethiopia. The second strategic plan (2009 to 2013) was developed to encompass integrated laboratory services.^7^ However, because of reforms within the Ethiopian health sector, the second strategic plan was revised in 2010 (2010 to 2015), to focus on three goals: establishing and strengthening laboratory quality systems, laboratory capacity building and laboratory accreditation.^8^ The Strengthening Laboratory Management Toward Accreditation (SLMTA) programme was selected in order to advance laboratory quality improvement and to expedite the accreditation preparation process.

SLMTA is a task-based, hands-on training programme aimed at effecting tangible laboratory improvements in developing countries.^9^ It includes a series of three workshops that are supplemented by assigned improvement projects and supportive site visits or mentoring.^10^ To evaluate its effect, an audit is performed before (baseline) and after (exit) SLMTA implementation using the World Health Organization Regional Office for Africa’s (WHO AFRO) Stepwise Laboratory Quality Improvement Process Towards Accreditation (SLIPTA) checklist.^11^ SLIPTA is an accreditation preparation framework that consists of an incremental recognition system awarding a range of zero to five stars, in contrast to the pass or fail score of traditional accreditation schemes. This stepwise approach recognises where a laboratory stands currently and encourages continual improvement through positive reinforcement by rewarding progress at each step.^3,4^

Laboratories in the public health system of Ethiopia are divided into four tiers: peripheral, hospital, regional reference and national reference laboratories.^7,12^ EPHI has national reference (multi-purpose) and research-only laboratories. Its nine national reference laboratories, each with different specialties, provide research, diagnostic, training and external quality assessment services. As per the administrative hierarchy, the higher-level laboratories support and oversee the next lower-level laboratories. For example, EPHI oversees the 12 regional reference laboratories, which oversee the 135 hospital laboratories, which, in turn, oversee health post/centre laboratories. However, in the case of new initiatives such as SLMTA, EPHI supports hospital laboratories until the regional reference laboratories are prepared to take over the task. This article evaluates the implementation and impact of the SLMTA programme in 45 laboratories in Ethiopia, as well as the role of supportive site visits and integration of selected improvement projects.

## Research methods and design

### Participating laboratories

A total of 45 laboratories were enrolled for participation in the SLMTA programme between 2010 and 2012 in order to facilitate quality improvement and to accelerate stepwise accreditation preparation. Laboratories were selected based on tier level, type, testing volume, geographical distribution and the operational area of the four American university-affiliated partners that participated in the audits and supportive site visits. Six national reference laboratories, seven regional reference laboratories and 32 public hospital laboratories were selected. For ease of implementation, laboratories were divided into two cohorts. National and regional laboratories were included in the first cohort from June 2010 to October 2011, along with 11 of the hospital laboratories; the remaining 21 hospital laboratories were included in the second cohort from January 2011 to May 2012 (Table 1).

**TABLE 1:** Timeline of SLMTA implementation in Ethiopia.

Activity	Cohort I (24 laboratories)	Cohort II (21 laboratories)
Baseline audit	June 2010	January 2011
First workshop	August 2010	March 2011
Improvement projects from the first workshop	4 improvement projects	4 improvement projects
Supportive site visits	2 visits	1 visit
Second workshop	December 2010	July 2011
Improvement projects from the second workshop	4 improvement projects	4 improvement projects
Supportive site visits	1 visit	None
Third workshop	March 2011	December 2011
Improvement projects from the third workshop	5 improvement projects	5 improvement projects
Supportive site visits	1 visit	None
Exit audit	October 2011	May 2012

SLMTA, Strengthening Laboratory Management Toward Accreditation.

### SLMTA implementation

The standard SLMTA model of three workshops was employed.^10^ The laboratory manager and quality officer, who are responsible for leading their staff and establishing a sustainable system for quality-assured laboratory services, were invited from each participating laboratory to attend the SLMTA training organised by EPHI in collaboration with the American Society for Clinical Pathology (ASCP). A total of 60 people were trained in each cohort. Trainings were facilitated by graduates of a two-week Training-of-Trainers course conducted by the African Centre for Integrated Laboratory Training in Johannesburg, South Africa, in collaboration with the US Centers for Disease Control and Prevention (CDC).^13^

### Improvement projects

Improvement projects were assigned based on the common gaps identified during the baseline audit and the components of the quality management system addressed during the specific training. Examples of areas in which improvement projects were conducted include customer satisfaction, external quality assessment, turnaround time and equipment utilisation rate. These improvement projects were expected to be implemented before the next training, or the exit audit in the case of improvement projects following the third workshop. Progress was reported to and monitored by EPHI. Additionally, participants were instructed to identify and assign more improvement projects to other staff members for execution.

### Supportive site visits

Supervisors trained in mentorship and supervisory skills were tasked to provide supportive site visits. Visits were scheduled approximately three weeks after each workshop. The plan required supervisors to spend two to three days in each SLMTA-enrolled laboratory. During this time, they conducted a site visit using prepared checklists that specifically focused on assigned improvement projects.

For cohort I, the supportive site visits were led by two or three supervisors; one from EPHI, one from an American university-affiliated partner and one (for three-person teams) from a regional reference laboratory. The four university partners were: Columbia University’s International Center for AIDS Care and Treatment Program in Ethiopia (ICAP-Ethiopia); the University of Washington’s International Training and Education Center on HIV in Ethiopia (ITECH-Ethiopia); the Johns Hopkins University Technical Support for the Ethiopian HIV/AIDS Initiative (JHU-TSEHAI); and the University of California, San Diego’s Technical Assistance Programme for HIV Prevention, Care and Treatment in Ethiopia (UCSD-Ethiopia). The university partners were assigned to specific regions covering the entire country. An average of 68 (range 24 to 80) hours of joint supportive site visits were provided to each of the first cohort laboratories.

Supportive site visits for cohort II did not take place as planned. The responsibility for providing supportive site visits was handed over to supervisors from the regional reference laboratories. Because of budget issues, no site visits occurred after the second or third workshop; total site visit time averaged two (range 0 to 24) hours per laboratory.

### Measuring improvements

Audits were conducted before (baseline) and after (exit) SLMTA implementation using the SLIPTA checklist. The pool of auditors comprised the experts who had conducted the supportive site visits. Audits were performed over two or three days by a group of two to three individuals who had not conducted supportive site visits or previous audits in the respective laboratory.

The audit checklist is based on 12 quality system components. Star levels were granted based on total audit scores; one, two, three, four and five stars were awarded for scores reaching 55%, 65%, 75%, 85% and 95%, respectively. One of the first cohort laboratories was unwilling to have an exit audit; thus results are presented for 44 of the 45 laboratories. Audit data were analysed using Microsoft^®^ Excel 2013. A paired *t*-test was used to compare baseline and exit results; a *t*-test for unequal variances was used to compare score changes between the two cohorts (*f*-test showed unequal variances, *p* < 0.01). Major success factors and challenges encountered during implementation of SLMTA were identified by focus group discussions.

## Results

**FIGURE 1:**
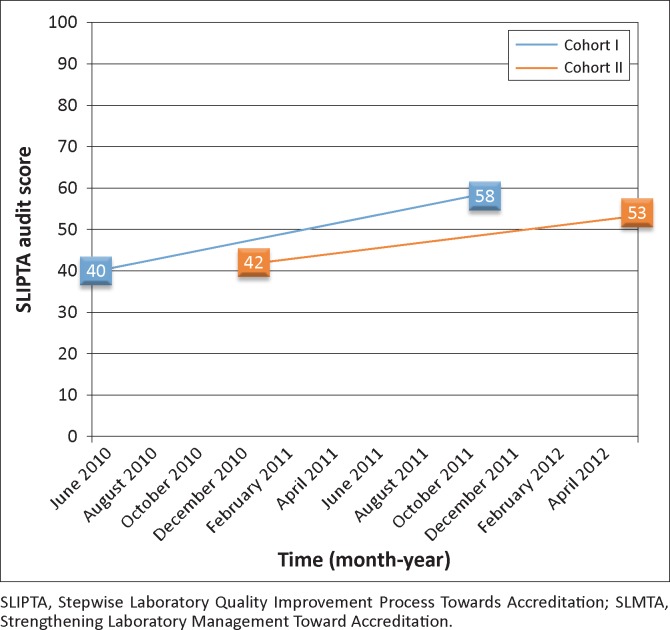
SLIPTA baseline and exit audit scores of the two SLMTA cohorts.

Overall, for the 44 laboratories that participated in both the baseline and exit audits, there was a 15 percentage point average increase in SLIPTA audit score, with 41% as the average baseline score and 56% the average exit score. For cohort I, average scores increased from 40% at baseline to 58% at exit audit (*p* < 0.01). The average scores of cohort II increased from 42% at baseline to 53% at exit audit (*p* < 0.01) (Figure 1).

Baseline scores ranged from 24% to 60% whereas exit scores ranged from 25% to 86%. Forty-two of the 44 laboratories (95%) improved their scores (from < 1 percentage point to 51 percentage points). A higher mean improvement was observed in cohort I laboratories (18 percentage points) compared with cohort II laboratories (11 percentage points), although the difference was of borderline statistical significance (*p* = 0.07). Five laboratories in cohort I showed an improvement of 30–51 percentage points, whilst no laboratories in cohort II achieved greater than a 30 percentage point improvement. However, decreases were also observed in two laboratories in cohort I (Table 2).

**TABLE 2:** TABLE 2: Improvement of laboratory SLIPTA scores.

Improvement (% points) (exit minus baseline)	Number of laboratories in which the improvement was observed		
	Cohort I^*^	Cohort II	Total
> 50	1	0	1
40 to 50	1	0	1
30 to 40	3	0	3
20 to 30	6	4	10
10 to 20	4	6	10
0 to 10	6	11	17
-4 to 0	2	0	2
**Total**	**23**	**21**	**44**

SLIPTA, Stepwise Laboratory Quality Improvement Process Towards Accreditation.

* Cohort I included a total of 24 laboratories; one laboratory with no exit audit was excluded from this analysis.

**FIGURE 2:**
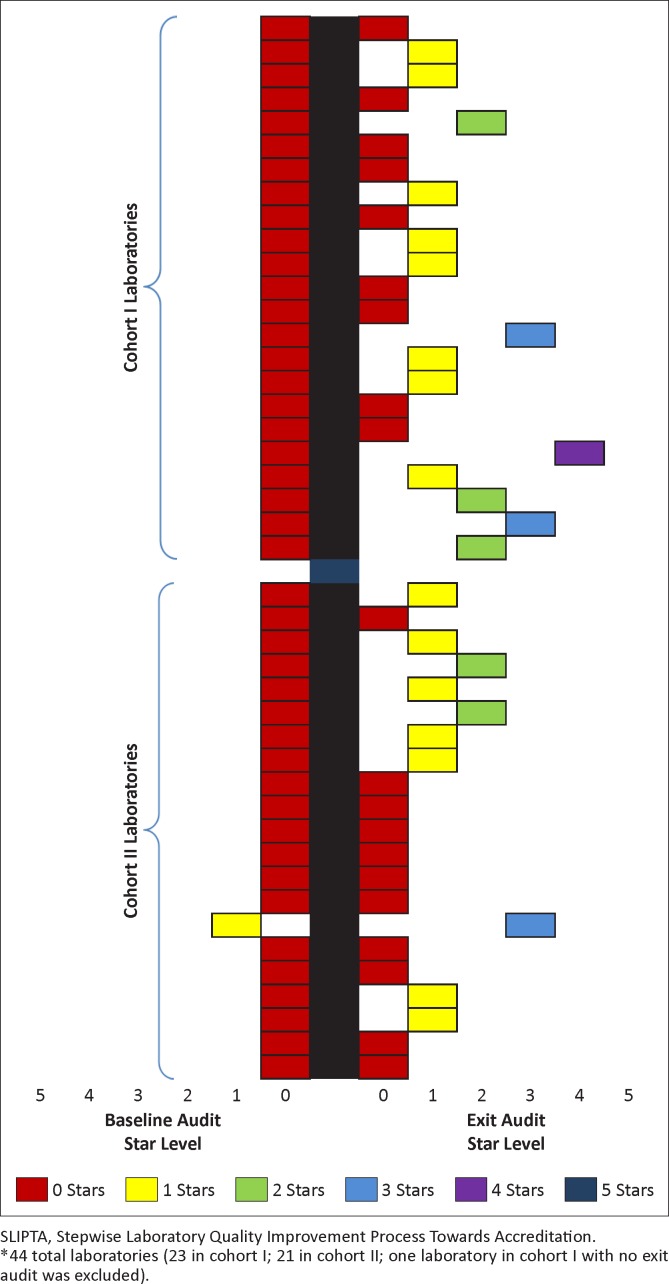
Change in SLIPTA star levels from baseline to exit audit.^*^

During the baseline audit, all laboratories in the first and all but one of the laboratories in the second cohort were at the zero-star level (scored < 55%). At the exit audit, 61% (*n* = 14/23) of the first and 48% (*n* = 10/21) of the second cohort laboratories attained one to four stars (Figure 2).

**FIGURE 3:**
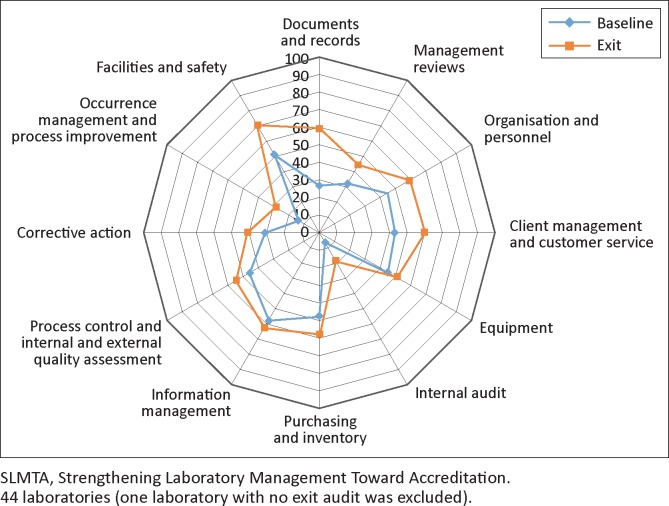
Percent compliance across the 12 quality system components of the 44 audited laboratories before (baseline) and after (exit) the implementation of SLMTA.

Results from the 12 quality system components of the SLIPTA audit checklist are presented in Figure 3. At both the baseline and exit audits, lower scores were observed for internal audit (6% baseline and 18% exit), occurrence management and process improvement (14% and 29%), corrective action (31% and 41%) and management reviews (32% and 44%). Relatively higher scores were shown in client management and customer service (42% and 59%), organisation and personnel (44% and 59%), information management (58% and 63%) and facilities and safety (51% and 71%). The greatest improvements were seen in the areas of documents and records (32 percentage points), facilities and safety (22 percentage points) and client management and customer service (17 percentage points). Smaller improvements were observed for information management (five percentage points), equipment (six percentage points) and process control and internal and external quality assessment (nine percentage points).

In focus group discussions, laboratory managers and quality officers pointed out several challenges to successful implementation of improvement projects. These challenges included: (1) poor awareness of the programme by upper management and regional health bureaus (i.e., SLMTA was not budgeted); (2) lack of harmonisation with other hospital improvement programmes; (3) inadequate awareness of quality management systems and insufficient commitment amongst non-SLMTA-trained staff; (4) high workload relative to available staff; (5) lack of quality manuals prescribing laboratory policies and procedures; and (6) inadequate supportive site visits. Detailed results from these focus group discussions are reported by Lulie et al.^14^

## Discussion

Prior to the introduction of SLMTA, several trainings and quality improvement initiatives had been implemented in hospitals and laboratories in Ethiopia, but little improvement was noted. By contrast, Ethiopian public health laboratories achieved remarkable improvements in quality systems after implementation of the SLMTA programme. Similar rapid and tangible improvements resulting from SLMTA training have been reported by other countries.^15,16^

Whilst similar training curricula and improvement projects were given to the two cohorts, a lower level of supportive site visits was administered to the second cohort. Results for the first cohort surpassed that of the second cohort, with nearly twice the improvement. Although not conclusive, these results suggest a positive effect of site visits. Site visits offer an opportunity to assess progress and enforce application of skills learned in the workshops. These visits often involve meetings with upper management and other staff members in order to elicit their support, which in turn motivates the implementers. Our data suggest that supportive site visits were critical with regard to reinforcing the knowledge and motivation offered during the training in order to achieve the expected behavioural changes required for quality improvement. On-site mentorship may also be used in a similar capacity; for example, it was found that after 10 weeks of dedicated on-site mentoring, laboratories in Lesotho demonstrated significant improvements.^15^

At baseline, participating laboratories displayed deficient quality management systems in many areas, as evidenced by all but one laboratory earning a zero-star rating. A poor laboratory quality management system facilitates erroneous or delayed results, which in turn can lead to poor or adverse patient outcomes.^17^ In Ethiopia, SLMTA implementation, combined with intensive supportive site visits, has proven to be a promising tool for the improvement of quality management systems.

Participating laboratories were found to be weaker in some quality system components than others. Specifically, internal audit, occurrence management and process improvement, corrective action and management reviews scored the lowest at both the baseline and exit audits. Similar results have been reported elsewhere.^16^ These weaker components are critical for continuous improvement of the overall quality system. Therefore, focused attention must be paid so that greater and sustainable advancements can be made.

Several barriers to the effective implementation of quality systems were identified during the focus group discussions. For example, an accreditation preparation budget was not included in the annual plans of the regional health bureaus, hospitals or laboratories. This omission prevented adequate supportive site visits for cohort II laboratories. It was also pointed out that promoting the importance of accreditation and the need for improving laboratory quality management systems is necessary for enhancing acceptance and ownership at all levels. Such promotion should be conducted prior to and during SLMTA implementation so as to ensure adequate support throughout the established healthcare structure and management teams.^18^

Hospital system and process reforms are underway in public health facilities throughout Ethiopia, with a goal of improving efficiency and effectiveness (i.e., high quality, low cost and rapid services), thereby increasing customer satisfaction.^14,19,20^ Strengthening laboratory quality management and moving toward accreditation should be considered an integral part of this process. Failure to implement laboratory quality management systems in harmony with other initiatives misses a valuable opportunity and could hinder the pace of progress and long-term sustainability of health system reforms and improvements.^18^ At the same time, the lack of laboratory quality manuals, guidelines, policies and procedures that provide clear and concrete directions and instructions posed a challenge during implementation. It was difficult for laboratories to execute quality improvement tasks consistently in the absence of these guiding materials.

Improvements were found to vary significantly amongst laboratories of each cohort; two laboratory exit scores showed a decrease from baseline, whilst the remaining 42 laboratory exit scores reflected an increase of up to 51 percentage points. The challenges identified during the trainings and focus group discussions could be contributing factors for this variation. Further collection and analysis of data throughout the implementation phase could enable programme leaders to identify and redress impeding factors.

### Recommendations

Improvements resulting from SLMTA implementation are encouraging and expanding SLMTA to other laboratories may help to improve quality management of laboratories for a better healthcare system. The evaluation suggests that supportive site visits are important for effective implementation of SLMTA and quality management systems. Availing a clear plan of supportive site visits and gaining upper management ownership in participating laboratories is needed in order to obtain the maximum benefit of SLMTA.

### Conclusion

Continuous advocacy at all levels of the health system will foster involvement of stakeholders and facilitate the integration of SLMTA with other hospital improvement initiatives, allowing SLMTA to benefit the system on a wider scale.

## References

[1] Panteghini M The future of laboratory medicine: Understanding the new pressures. Clin Biochem Rev. 2004;25(4):207–215. 18458714 PMC1934959

[2] Wians FH Clinical laboratory tests: Which, why, and what do the results mean? Lab Medicine. 2009;40:105–113. http://dx.doi.org/10.1309/LM4O4L0HHUTWWUDD

[3] Gershy-Damet GM, Rotz P, Cross D, et al The World Health Organization African region laboratory accreditation process: Improving the quality of laboratory systems in the African region. Am J Clin Pathol. 2010;134(3):393–400. http://dx.doi.org/10.1309/AJCPTUUC2V1WJQBM 20716795 10.1309/AJCPTUUC2V1WJQBM

[4] Nkengasong JN A shifting paradigm in strengthening laboratory health systems for global health: acting now, acting collectively, but acting diifferently. Am J Clin Pathol. 2010;134(3):359–360. http://dx.doi.org/10.1309/AJCPY5ASUEJYQ5RK 20716789 10.1309/AJCPY5ASUEJYQ5RK

[5] Vitoria M, Granich R, Gilks CF, et al The global fight against HIV/AIDS, tuberculosis, and malaria: Current status and future perspectives. Am J Clin Pathol. 2009;131(6):844–848. http://dx.doi.org/10.1309/AJCP5XHDB1PNAEYT 19461091 10.1309/AJCP5XHDB1PNAEYT

[6] Nkengasong JN, Nsubuga P, Nwanyanwu O, et al Laboratory systems and services are critical in global health: Time to end the neglect? Am J Clin Pathol. 2010;134(3):368–373. http://dx.doi.org/10.1309/AJCPMPSINQ9BRMU6 20716791 10.1309/AJCPMPSINQ9BRMU6 PMC7109802

[7] Ethiopian Health and Nutrition Research Institute Master plan for the public health laboratory system in Ethiopia, Second Edition (2009–2013). Addis Ababa: EHNRI; 2009.

[8] Ethiopian Health and Nutrition Research Institute Five year, balanced score card-based strategic plan (2010–2015). Addis Ababa: EHNRI; 2010.

[9] Yao K, McKinney B, Murphy A, et al Improving quality management systems of laboratories in developing countries: An innovative training approach to accelerate laboratory accreditation. Am J Clin Pathol. 2010;134(3):401–409. http://dx.doi.org/10.1309/AJCPNBBL53FWUIQJ 20716796 10.1309/AJCPNBBL53FWUIQJ

[10] Yao K, Maruta T, Luman ET, Nkengasong JN The SLMTA programme: Transforming the laboratory landscape in developing countries. Afr J Lab Med. 2014;3(2), Art. #194, 8 pages. http://dx.doi.org/10.4102/ajlm.v3i1.194 10.4102/ajlm.v3i2.194 PMC4703335 26752335

[11] World Health Organization’s Regional Office for Africa WHO AFRO Stepwise Laboratory Quality Improvement Process Towards Accreditation (SLIPTA) Checklist [document on the Internet]. c2012 [cited 2014 May 31]. Available from: http://www.afro.who.int/en/clusters-a-programmes/hss/blood-safety-laboratories-a-health-technology/blt-highlights/3859-who-guide-for-the-stepwise-laboratory-improvement-process-towards-accreditation-in-the-african-region-with-checklist.html

[12] World Health Organization’s Regional Office for Africa (WHO AFRO) Consultation on technical and operational recommendations for clinical laboratory testing harmonization and standardization. Maputo: WHO AFRO; 2008.

[13] Maruta T, Yao K, Ndlovu N, Moyo S Training-of-trainers: A strategy to build country capacity for SLMTA expansion and sustainability. Afr J Lab Med. 2014;3(2), Art. #196, 7 pages. http://dx.doi.org/10.4102/ajlm.v3i2.196 10.4102/ajlm.v3i2.196 PMC4703333 26753131

[14] Lulie AD, Hiwotu TM, Mulugeta A, et al Perceptions and attitudes toward SLMTA amongst laboratory and hospital professionals in Ethiopia. Afr J Lab Med. 2014;3(2), Art. #233. http://dx.doi.org/10.4102/ajlm.v3i2.233 10.4102/ajlm.v3i2.233 PMC5637786 29043195

[15] Mothabeng D, Maruta T, Lebina M, et al Strengthening Laboratory Management Towards Accreditation: The Lesotho experience. Afr J Lab Med. 2012;1(1), Art. #9, 7 pages. http://dx.doi:10.4102/ajlm.v1i1.9 10.4102/ajlm.v1i1.9 PMC5644518 29062729

[16] Luman ET, Yao K, Nkengasong JN A comprehensive review of the SLMTA literature part 2: Measuring success. Afr J Lab Med. 2014;3(2), Art. #276, 8 pages. http://dx.doi.org/10.4102/ajlm.v3i2.276 10.4102/ajlm.v3i2.276 PMC5637800 29043201

[17] Peter TF, Rotz PD, Blair DH, et al Impact of laboratory accreditation on patient care and the health system. Am J Clin Pathol. 2010;134(4):550–555. http://dx.doi.org/10.1309/AJCPH1SKQ1HNWGHF 20855635 10.1309/AJCPH1SKQ1HNWGHF

[18] Silva P Guidelines for establishment of accreditation of health laboratories [document on the Internet]. c2007 [cited 2014 May 31]. Available from: http://cedoc.cies.edu.ni/general/World%20Health%20Organization%20HIV-AI%20(D)2/pdfs/Publications_SEA-HLM-394.pdf

[19] Bradley E, Hartwig KA, Rowe LA, et al Hospital quality improvement in Ethiopia: A partnership-mentoring model. Int J Qual Health Care. 2008;20(6):392–399. http://dx.doi.org/10.1093/intqhc/mzn042 18784268 10.1093/intqhc/mzn042

[20] Federal Democratic Republic of Ethiopia, Ministry of Health Annual performance report of HSDP-III [document on the Internet]. c2009. [cited 2014 Oct 03]. Available from: http://www.moh.gov.et/documents/26765/28899/Annual+Perfomance+Report+2009/a6c0fcff-d2f5-4c2b-b9d1-1eac2c0a7531;jsessionid=B39C317151AA1D3CF8153A46CE48FAA4?version=1.1

